# Physical Activity and Daily Routine among Children Aged 0–12 during the COVID-19 Pandemic in Spain

**DOI:** 10.3390/ijerph18020703

**Published:** 2021-01-15

**Authors:** Javier Cachón-Zagalaz, M.ª Luisa Zagalaz-Sánchez, Víctor Arufe-Giráldez, Alberto Sanmiguel-Rodríguez, Gabriel González-Valero

**Affiliations:** 1 Department of Didactics of Musical, Plastic and Body Expression, Faculty of Humanities and Educational Sciences, University of Jaén, 23071 Jaén, Spain; jcachon@ujaen.es (J.C.-Z.); lzagalaz@ujaen.es (M.L.Z.-S.); 2 Specific Didactics Department, Research and Diagnostic Methods in Education, Education Faculty, University of a Coruña, 15001 Coruña, Spain; v.arufe@udc.es; 3 Faculty of Language and Education, University of Antonio de Nebrija, 28015 Madrid, Spain; asrgz2014@gmail.com; 4 Faculty of Language and Education, University of Camilo José Cela, 28692 Madrid, Spain

**Keywords:** COVID-19, children, lockdown, physical activity, daily activities

## Abstract

The COVID-19 pandemic has impacted the lives of many people. Objective: The aim of the present study was to analyse the physical activity (PA) and daily routine among children (0–12 years) during lockdown and to establish the main relationships among the variables. Methods: A quantitative study with a descriptive–comparative and cross-sectional design carried out. The sample, selected for convenience, consisted of 837 Spanish children aged 0–12. The “Children and confinement” questionnaire was used, distributed electronically through Google Forms and social networks and activated for 45 days. The individuals participating in this study were mainly children (50.2%) who were in primary education (44.8%). Predominantly, the families of participants were biparental (87.9%), with established routines and schedules (85.7%). Results and conclusions: The use of digital screens is an important part of children’s daily routine. Their daily activities were practiced for more than three–six years, with more by girls. The time children devoted to sleep was directly proportional to the time they devoted to physical activity and indirectly proportional to the time they spent watching screens. The children who slept the most were those aged zero–three years, especially girls, who belonged to large families. The levels of physical activity in the sample were low, as were the times spent on activities such as music or games.

## 1. Introduction

Physical activity (PA) constitutes one of the fundamental pillars for the correct physical and psychological developments of a child. Furthermore, it is considered an essential means for the prevention of pathologies, improvement of health and can contribute to reducing childhood obesity [1,2,3]. Thus, childhood should be seen as a critical time for promoting healthy behaviours insofar as PA and energy balance [4] and can impact multiple health and developmental outcomes, including psychosocial well-being [5]. According to Hinkley et al. [5], psychosocial well-being is vital for children’s school readiness and future mental health.

During the confinement in Spain, the activities that children could perform on a regular basis became altered. In particular, practicing the recommended amount of physical activity was made more difficult by the closing of sports facilities and parks [6].

Lockdowns can be more harmful for those who share a home with many live-in partners. Therefore, Rolla [7] recommended spending more time outdoors as a way of mitigating the high rate of overcrowding among the most disadvantaged homes in Spain. Therefore, children can interpret lockdown and the threat of COVID-19 as a danger that causes them anxiety. Even children under two years of age have noticed the changes in their lives and the possible absences of caregivers (grandparents). It is important to address these states of anxiety [8] and the psychological impacts of the quarantine [9]. In recent months, 72% of children and 77% of adults have reported a worsening of their emotional health, and 55% of families have reported an increase in parent–child conflicts at home [10].

Currently, physical exercise (PE) classes at school are the only spaces where children and youngsters can do compulsory physical activity. It should be a space for body movement, fun and gratifying emotions [11], as this subject influences many aspects of their daily lives [12]. Above all, PA contributes enormously to healthy behaviours, and the three-month lockdown prevented children from exercising sufficiently [6], which was associated with weight gain [13]. The WHO [14,15] indicates that children and youngsters aged 5–17 years should invest in at least 60 min per day of moderate-to-vigorous PA.

To alleviate the effects of the pandemic and lockdown on children’s learning, Save the Children created the programme “At your side”. According to this nongovernmental organisation (NGO) [10], the digital divide has affected 39% of children, and 64% have found it difficult to keep up with their schooling due to a lack of support by adults. Guan et al. [16] also provided recommendations for daily PA time to counteract the effects of lockdown and the increased use of digital technologies. These authors gave recommendations to parents and caregivers for the promotion of healthy daily behaviours. Referring specifically to crises in the Spanish education system caused by COVID-19, Gómez-Gerdel [17] mentioned some advantages to virtual education, namely the possibility for children to acquire greater autonomy in daily household tasks and to improve family relations by spending more time together [18].

The WHO [19] and other researchers [20] have warned against screen abuse and physical inactivity in early childhood. Although the excessive use of Information and Communication Technologies (ICTs) by children is nothing new [21], the lockdown has compounded the effect in children and adults [22]. Since computer use and Internet access are practically universal, many researchers [23,24,25,26,27,28,29,30,31] have proposed prevention programmes and instruments to detect addictions and other behaviour problems. To improve the situation, a healthy mix of lifestyle behaviours in childhood involving less screen time, healthy eating patterns and balanced daily PA, is endorsed [32].

Daily routines are an important aspect of childhood development. These include activities such as homework [33,34], participation in domestic chores [35,36,37,38], reading and learning to play a musical instrument [39,40]. Family- or parent-led play can be particularly influential [41]. Some studies have reported a clear association between families’ levels of happiness and time spent playing with children [42]. Free play is also important in promoting skills such as thinking and creativity and can predict social success in adulthood, especially if there is an optimal amount of PA [43,44].

The time spent away from school will have a greater impact in grades where children have to achieve an important learning outcome, such as reading and writing in the last year of preschool [45]. Nevertheless, sociologists and educationalists assure that the youthfulness of these children and the good work of their teachers will allow them to recover the knowledge missing during lockdown in a short time.

Only when the pandemic ends will it be possible to evaluate the total health and social, educational and economic impacts [46] in terms of negative changes in the lifestyles of Spanish children during the COVID-19 lockdown (from 15 March to 2 June) and aggravated by the long period of time without attending school. The aim of the present study was to analyse the PA and daily routines carried out by the child population (0–12 years) during the lockdown, as well as to establish the main relationships among the variables. We hypothesised that PA at home during lockdown would be lower than usual, that healthy behaviours would be affected by the lack of movement and that daily routines would primarily be inclined toward using digital screen devices.

## 2. Materials and Methods

### 2.1. Design and Participants

Our quantitative study was carried out using a descriptive, comparative and cross-sectional design, in which a single measurement was made for a single group. The sample was composed of 837 Spanish children aged 0–12 years (M = 6.22; SD = 3.36). The selection of the participants was done by convenience; families with children in the infant and primary education stages during the COVID-19 pandemic were invited to participate, and we had easy access to them. The distribution of the sample was homogeneous, represented by 50.2% for boys (*n* = 420) and 49.8% for girls (*n* = 417)—in total, 837 children of school-going ages from 0 to 12 years old. Table 1 shows the distribution of the sample according to the region (Comunidad Autónoma) in Spain.

### 2.2. Variables and Instruments

Sociodemographic variables. For this variable, an ad hoc questionnaire was used, which was prepared by us and in which we recorded the sex (categorised as “boy” or “girl”); age; type of family (categorised as “biparental”, “single parent”, “numerous family” or “biparental with separation”) and the establishment of routines and schedules in the children (categorised as “yes” or “no”). These categories were created on the basis of the main characteristics collected by the National Institute of Statistics (INE). This questionnaire is similar to the already existing ones and forms the basis for the very specific data that are collected by any instrument of these characteristics, so it does not need validation. However, the questions formulated were validated by expert judgement and served as a pilot study to provide a solution to the current problem. In addition, various items of the questionnaire created ad hoc were extracted from the validated questionnaire on Equipment and Use of Information and Communication Technologies in Homes (TIC-H2019) prepared by the National Institute of Statistics (INE) following the recommendations of the Statistical Office of the European Union (EUROSTAT), from which items related to the use of technological devices were extracted.

Healthy behaviours. This variable was recorded in order to know and count the time that children performed sedentary behaviours, such as the use of digital screens (time in front of video consoles, televisions, computers, tablets and mobile phones). This data was collected in minutes. The hours dedicated to sleep and the sum of the time spent on PA during lockdown were recorded, being in turn categorised from “does not practice” to “practices 6 to 7 times per week”.

Daily activities during lockdown. This variable refers to the activities usually carried out by the children at home during the lockdown period, such as artistic work, school and domestic chores, free and family play, listening to music or dancing and/or reading. The sum of the time spent by the child in these types of activities during the period of lockdown was recorded in minutes.

### 2.3. Procedure

The “Children and Lockdown” questionnaire was developed by seven experts from the fields of general didactics, psychology, body expression and sport. After checking the data and the assessment of the questions, a 93% correlation rate was obtained. This was calculated by observing the experts’ findings, dividing it by the total number of items and multiplying by 100. The questionnaire was created on the Google Forms platform and disseminated through social networks to reach the population under investigation, parents with children from 0 to 12 years of age, because children are not trained to give the necessary answers, especially the youngest ones. Likewise, contacts of various education professionals who had access to a large number of families were used to ensure that the questionnaire was well-distributed in the different autonomous communities.

The questionnaire was activated for 45 days (from 23 March to 6 May 2020) within the lockdown period established by the Spanish government [47]. Of all the questionnaires received in the database, 76 were eliminated, because they were not correctly completed or belonged to another educational stage.

By completing the form, all the participants gave their consent to anonymously work with the data. Throughout the research, the ethical principles reflected in different official documents and treaties on research ethics were taken into account, thus guaranteeing the anonymity of the participants, the confidentiality of the data reflected in the questionnaire and other ethical considerations related to educational research [48,49].

### 2.4. Data Analysis

A descriptive analysis was used to determine the sociodemographic characteristics and behaviours during the COVID-19 pandemic using the mean deviation (M), standard deviation (SD) and frequencies (%). We also used the Kolmogorov-Smirnov test to determine the normality and homogeneity of the variances in the variables. To establish the differences between the variables, we used the Student’s *t*-test for independent samples and the one-factor ANOVA, employing Pearson’s chi-square statistical indicator to verify such differences. Pearson’s bivariate correlations were thus used to establish relationships between the parameters with mean values at the significance levels of *p* = 0.01 and *p* = 0.05. The statistical software SPSS 25.0 (IBM Corp, Armonk, NY, USA) was used for data analysis and processing.

### 2.5. Ethical Aspects

By completing the form, all participants gave their consent to work anonymously with the data. Throughout the research, the ethical principles reflected in different documents and official treaties on research ethics were taken into account, guaranteeing the anonymity of the participants, the confidentiality of the data reflected in the questionnaire and other ethical considerations related to educational research [48].

## 3. Results

Table 2 shows the main characteristics of the study sample. The majority were male (50.2%) and represented the primary education stage (44.8%). The predominant type of family was the two-parent family (87.9%), who highlighted that routines and schedules were established to perform tasks (85.7%), although the highest percentage of children did not perform PA for six days a week in lockdown (34.8%) but was very close to those who practiced it between two and three days a week.

Table 3 presents children’s healthy, everyday behaviours related to their sex and the establishment of routines and schedules for tasks. Statistically significant results were obtained from both relationships (*p* ≤ 0.05). Girls slept more hours a day (M = 10.04; SD = 1.53) and spent more time on daily activities (M = 428.22; SD = 185.24). However, boys spent more time in front of digital screens (M = 163.29; SD = 125.27) compared to girls (M = 152.05; SD = 124.45). Families that did establish routines and schedules for performing tasks were associated with more time spent practicing daily PA (M = 38.13; SD = 35.01). However, those who did not establish them showed a greater number of hours sleeping (M = 10.32; SD = 1.64) and in front of digital screens (M = 186; SD = 135.20).

Table 4 shows children’s healthy, everyday behaviours in relation to age and family type. With respect to age (*p* ≤ 0.05), higher average values were observed in primary education (6–12 years) for daily PA practice (M = 38.95; SD = 30.25) and the use of digital screens (M = 213.78; SD = 133.12). The highest parameters for daily activities (M = 441.83; SD = 160.48) were in the second cycle of infant education and, for sleep hours (M = 10.98; SD = 1.82), in the first cycle of that stage. According to the type of family (*p* ≤ 0.05), a greater use of screens was shown in two-parent families who are separated (M = 192.65; SD = 105.05), more hours of sleeping in large families (M = 10.66; SD = 1.32) and daily activities in single-parent families (M = 510.13; SD = 207.49).

Table 5 shows the results related to PA practice during lockdown as a function of children’s healthy and daily behaviours, for which statistically significant data were obtained (*p* ≤ 0.05). It was confirmed that those who spent more time on PA were those who practiced it six to seven days/week (M = 75.09; SD = 58.65), as opposed to the use of digital screens; those who did not practice PA spent more time on them (M = 173.09; SD = 115.56). Similarly, those who recorded the most hours of sleep (M = 10.19; SD = 0.57) and spent the most time on daily activities (M = 476.52; SD = 180.50) were those who performed PA six to seven days/week.

Table 6 relates healthy, everyday behaviours with the age of the participants. During lockdown, a direct and positive correlation was established between age with daily PA practice (r = 0.168 *) and the use of digital screens (r = 0.595 **), with the relationship with daily sleep hours being indirect and negative (r = −0.547 **), the latter two (digital screens and sleep hours) registering a mean relationship strength. Thus, sleeping hours were indirectly associated with the use of digital screens (r = −0.395 **). Finally, daily activities during lockdown were directly associated with the use of digital screens (r = 0.234 **) and daily PA practice (r = 0.301 **).

## 4. Discussion

In this sense, teachers need data that allow them to develop the education of students—in this case, in infant and primary education—and for this, we investigated how the pandemic has influenced the activities of Spanish children from zero to 12 years, such as PA; healthy behaviours and daily activities (artistic work, school and home chores, free play and family, listening to music or dance and/or reading).

Five months without physically attending school carries the risk that the objectives established for each year will not be met; this will have to be checked and, if necessary, corrected to ensure that the right to education of school-going children prevails. A partnership between the government and academic communities can be a key component of the public health response to an emergency, such as the COVID-19 pandemic, in which we agree with Cruz-Correa et al. [50].

We know and proved in this study that PE influences many aspects of children’s daily lives [12] and contributes to the acquisition or improvement of healthy behaviours and, therefore, in physical and emotional development. A lack of PA is associated with obesity and other health problems [13]. This was expressed by the WHO [14,15] and was taken up by Guan et al. [16] when they praised the importance of PA to avoid child sedentarism as an effect of lockdown. They indicated that data from the pre-COVID-19 period showed that, on average, only a fifth of preschoolers and less than 10% of school-aged children met all PA and movement guidelines. According to other researchers [51], low percentages of PA in preschool-aged children have been found. Similarly, Ji et al. [52] indicated that Chinese preschool students performed little PA. Although Arufe et al. [53] believed that children under 12 years of age performed a minimum amount of PA with friends in sports schools in times of lockdown, the activities performed at home increased.

Likewise, Gómez-Gerdel [17] talked about the crisis in the Spanish educational system originated by COVID-19 and virtual education, something that contradicts our results, as children spend many hours using digital screens and parents complain about not knowing how well online teaching works, despite the fact there are programs to help families by Save the Children [10] and “A tu lado”, for example. In contrast, some authors, such as Navarrete et al. [54], believe that technological tools also help and support the teaching–learning process for people with disabilities, favouring inclusion.

We tried to find out how long children engage in sedentary behaviours with the manipulation of digital screens (sitting in front of game consoles, televisions, computers, tablets and mobile phones), despite the fact that, sometimes, playing with a mobile phone for 10 to 20 min can affect their balance and cause dizziness [55]. We compared this usage with the hours of sleep and the time spent performing PA (from no practice to six to seven times practicing a week).

This use and/or abuse of digital screens has become one of the daily activities that appears in our study counteracted by PA [19,20]. The problem is that it can become an addiction or behavioural complication [23,24,25,26,27,28,29,30,31]. The use of screens [21], together with schoolwork [33,34], domestic occupations [35,36,37,38] and reading habits [56,57], make up the usual tasks of children during lockdown. Families who had established routines and schedules for performing them obtained better results than those who did not. In South Korea, 81% of parents reported that their children’s sedentary screen times increased during the time of confinement [16]. In another study [22], they showed that Spanish children’s exposure to screens was 15 h a week and that their lifestyles fell within the health standards in terms of hours of rest and PA habits. The results of another investigation [58] showed that sedentary leisure (number of hours of television, computer and console) maintained a significant and inverse relationship with hours of sleep and PA.

In relation to sleep, girls sleep more hours a day (first cycle of infant education, of course) than boys and spend more time on daily activities (second cycle of infant education). Those who recorded the most hours of sleep and spent the most time on daily activities were those who performed PA (six to seven days). According to other authors [59], children slept for 9.72 h per night. The duration of sleep the previous night was positively associated with daytime PA. Other authors [60] indicated that a longer sleep duration was generally associated with better body composition, emotional self-regulation and growth in children aged 0–4 years. Guan et al. [16], in interviews with parents of preschool-aged children in Beijing (China), found that, compared to pre-COVID-19, almost all children went to bed later and woke up later.

With respect to music, although playing an instrument improves cognitive capacity [39,40], we saw that, on the days of lockdown, the children practiced nursery rhymes and listened to music and danced, turning this action into a PA. Likewise, play, sometimes related to PA [44], has taken up much of children’s time as a replacement for using technological devices [41], thus reinforcing parent–child relationships and increasing the happiness of parents and children [43].

The most significant differences that will be established in education, after this health and social crisis, will be found in the change of the cycles (last year of infant and first of primary and sixth of primary, whose students will go on to Secondary Education centres), although only when the pandemic ends will we be able to evaluate the total health, social, educational and economic impacts of this global disaster [46] in consideration of the negative changes in the lifestyles of Spanish children during lockdown. Other researchers from other countries have also tried to measure the impacts of confinement on levels of PA and sedentary behaviours. Thus, the study by Genevieve et al. [6] suggested that U.S. children performed less PA and engaged in more sedentary behaviours during the early-COVID-19 period as compared to before the pandemic. The most common physical activities during the early-COVID-19 period were free play or unstructured activities such as running and walking. Children were more likely to perform PA at home indoors or on neighbourhood streets during the periods of lockdown against before COVID-19. A sedentary lifestyle can become ingrained, leading to an increased risk of obesity, diabetes and cardiovascular disease in children. Following this idea, another study [61] evidenced the negative impacts of the COVID-19 confinement on the PA levels and sedentary behaviours of Spanish children. These findings should be taken into account to design and implement public health strategies for preserving children’s health during and after the pandemic, particularly for children with social vulnerabilities. A study carried out with 2159 Portuguese children [62] also confirmed less time devoted to PA during confinement compared to what was generally reported on normal days and highlighted the need to establish strategies to increase the levels of PA of children in families whose parents are working and have no outdoor space.

Therefore, in our sample, the hypothesis that PA performed at home would be low was confirmed, because low levels of PA were recorded. The results were similar to other studies cited above. On the other hand, the initial hypothesis that children would spend a large part of their time at home using electronic screen devices was also fulfilled, with high levels of time spent on different electronic devices.

## 5. Conclusions

Most of the participants in this study were children at elementary school from two-parent families who had established routines and schedules to perform tasks. The children in these families spent more time practicing PA on a daily basis compared to those without rules, who slept more and used digital screens more.

A large percentage of children performed PA during the confinement, especially elementary school children, but they recorded little PA time. The use of digital screens has also formed a prominent part of daily activities to a greater extent among primary school children. Daily activities were practiced more by the between three to six years old group and by girls. Time spent sleeping was directly proportional to the time spent in PA and indirectly proportional to the time spent using display devices. The children who slept the most were those from zero to three years old and those of other ages, especially girls, who belonged to large families.

PA is closely related to healthy behaviours and is part of many daily activities for children ages 0–12, including those related to music and play. During the confinement and the following days, the use of digital screens was the most performed activity, followed with the least amount of time in minutes dedicated to PA in the child population (0–12 years). In this sense, PA minimally counteracts a sedentary lifestyle of the use of screens. It would be useful to analyse in the future daily activities individually so that we can observe the time that children dedicate to each of the activities and, therefore, their interests.

The strengths of this study are its timeliness and punctuality, as it was carried out during the initial dates of the lockdown, providing educational perspectives that other studies do not capture by focusing only on health issues. It is also recommended for future research to create and validate a questionnaire that allows measuring different variables related to the lifestyles of children during the period of confinement in order to be able to use the same data collection instrument for all researchers who work in the future in studies on this subject.

## 6. Study Limitations

This study was not without its limitations. Although the questionnaire was created on the basis of various national questions and was constructed by experts in the field of PA and education, no quantitative validation was carried out. Thus, the reliability of the study was based on the percentage of concordance of the experts who judged the questionnaire. Likewise, the self-informed opinion of the families could have been another limitation; however, during the pandemic, it was the method used due to the principle of opportunism and the need to offer solutions to the situation. It should be noted that there were also limitations in the statistical analysis of the study; however, this helped to raise some prospects for the future. Firstly, there are other statistical methods that emphasise the importance of the relationships between variables and also can establish a statistical analysis of covariances or structural equation models. In addition, and although the questionnaire is accessible to the entire scientific community, the results will surely be uploaded to a data cloud. However, the importance of this study and its practical applications should be stressed, as it will provide public administration and educational centres with relevant information on children aged 0–12, with the purpose of promoting their healthy physical conditions and intervening in the problem of sedentarism and in other psychosocial aspects.

## Figures and Tables

**Table 1:** Distribution of the sample according to the region in Spain.

Region (Comunidad Autónoma)	Frequency (*n*)	Percentage (%)
Andalucía	249	29.7
Galicia	244	29.2
Valencia	67	8.0
Madrid	50	6.0
Castilla and León	36	4.3
Baleares	28	3.3
País Vasco	27	3.2
Canarias	26	3.1
Aragón	22	2.6
Murcia	18	2.2
Cataluña	16	1.9
La Rioja	15	1.8
Asturias	12	1.4
Cantabria	8	1.0
Extremadura	7	0.8
Castilla and La Mancha	6	0.7
Navarra	6	0.7
Total	837	100.0

**Table 2:** Characteristics of the sample.

Characteristics	Category	Frequency (*n*)	Percentage (%)
Sex	Boy	420	50.2%
	Girl	417	49.8%
Age	First cycle of Infant School (0–2 year)	202	24.1%
	Second cycle of Infant School (3–5 year)	260	31.1%
	Primary Education (6–12 year)	375	44.8%
Family type	Biparental	736	87.9%
	Single parent	34	4.1%
	Numerous family	9	1.1%
	Biparental with separation	58	6.9%
Routines and task schedules	Yes	717	85.7%
	No	120	14.3%
PA during lockdown	No	291	34.8%
	2 to 3 days/week	273	32.6%
	4 to 5 days/week	164	19.6%
	6 to 7 days/week	109	13.0%
		**Media (M)**	**SD**
Healthy behaviour	Daily Physical Activity	36.47	35.46
	Daily use digital screens	161.18	124.80
	Hours of sleep per day	9.93	1.51
Daily activities	Daily activities	413.81	185.31

**Table 3:** Sex and the establishment of routine in reference to children’s healthy and daily behaviours.

					Levene Test		*T*-Test	
Characteristics	Category		M	SD	F	Sig	*t*	Sig
By sex	Daily Physical Activity	Boy	38.00	40.695	7.583	0.006	1.255	0.210
		Girl	34.93	29.242				
	Daily use digital screens	Boy	163.29	125.27	0.165	0.799	2.491	0.013
		Girl	152.05	124.45				
	Hours of sleep per day	Boy	9.82	1.48	0.022	0.882	−2.135	0.033
		Girl	10.04	1.53				
	Daily activities	Boy	399.51	184.49	0.101	0.750	−2.246	0.025
		Girl	428.22	185.24				
Routines and task schedules	Daily Physical Activity	Yes	38.13	35.01	0.140	0.708	3.323	0.001
		No	26.58	36.68				
	Daily use of digital screens	Yes	160.37	123.06	1.518	0.218	−0.457	0.038
		No	186.00	135.20				
	Hours of sleep per day	Yes	9.86	1.48	4.089	0.043	−3.075	0.005
		No	10.32	1.64				
	Daily activities	Yes	412.86	177.29	8.998	0.003	−0.361	0.363
		No	419.47	228.26				

Note: Mean (M) and standard deviation (SD).

**Table 4:** Age and type of family according to children’s healthy and everyday behaviours.

Characteristics	Category		M	SD	F	*p*-Value
Age	Daily Physical Activity	First cycle of Infant School (0–2 year)	30.86	42.59	3.534	0.030
		Second cycle of infant school (3–5 year)	37.26	36.01		
		Primary Education (6–12 year)	38.95	30.25		
	Daily use digital screens	First cycle of Infant School (0–2 year)	85.54	99.55	88.000	0.000
		Second cycle of infant school (3–5 year)	144.07	90.75		
		Primary Education (6–12 year)	213.78	133.12		
	Hours of sleep per day	First cycle of Infant School (0–2 year)	10.98	1.82	94.067	0.000
		Second cycle of infant school (3–5 year)	9.95	1.28		
		Primary Education (6–12 year)	9.34	1.11		
	Daily activities	First cycle of Infant School (0–2 year)	381.36	225.49	6.164	0.002
		Second cycle of infant school (3–5 year)	441.83	160.48		
		Primary Education (6–12 year)	411.87	174.59		
Family type	Daily Physical Activity	Biparental	36.94	36.582	0.509	0.677
		Single parent	34.91	28.245		
		Numerous family	33.33	31.623		
		Biparental with separation	29.76	19.526		
	Daily use digital screens	Biparental	159.16	126.42	2.838	0.043
		Single parent	192.65	105.05		
		Numerous family	140.00	113.99		
		Biparental with separation	173.79	123.42		
	Hours of sleep per day	Biparental	9.97	1.54	3.142	0.025
		Single parent	9.44	0.90		
		Numerous family	10.66	1.32		
		Biparental with separation	9.70	1.62		
	Daily activities	Biparental	407.09	183.73	3.827	0.010
		Single parent	438.86	188.61		
		Numerous family	438.11	86.08		
		Biparental with separation	510.13	207.49		

**Table 5:** Physical activity during lockdown according to children’s healthy, everyday behaviours.

Characteristics	Category		M	SD	F	X^2^
PA during lockdown	Daily Physical Activity	No	12.40	17.69	150.116	0.000
		2 to 3 days/week	37.63	19.83		
		4 to 5 days/week	51.59	26.36		
		6 to 7 days/week	75.09	58.65		
	Daily use digital screens	No	173.09	115.56	4.628	0.018
		2 to 3 days/week	165.68	147.85		
		4 to 5 days/week	157.09	108.36		
		6 to 7 days/week	141.50	106.97		
	Hours of sleep per day	No	10.16	1.73	7.610	0.000
		2 to 3 days/week	9.80	1.36		
		4 to 5 days/week	9.56	1.14		
		6 to 7 days/week	10.19	1.57		
	Daily activities	No	390.77	215.34	6.376	0.000
		2 to 3 days/week	422.69	166.60		
		4 to 5 days/week	398.24	147.53		
		6 to 7 days/week	476.52	180.50		

**Table 6:** The correlation of healthy, everyday behaviours and ages of the children.

	Age	DPA	UDDS	HSPD	DA
**Age**	1	0.168 *	0.595 **	−0.547 **	0.146
**DPA**		1	−0.118	−0.131	0.301 **
**UDDS**			1	−0.395 **	0.234 **
**HSPD**				1	−0.111
**DA**					1

Note 1: Daily Physical Activity (DPA), Daily use of digital screens (UDDS), Hours of Sleep Per Day (HSPD) and Daily Activities (DA). Note 2: Correlation is significant at the 0.01 level (**), and correlation is significant at the 0.05 level (*).

## Data Availability

Data sharing not applicable.
